# Contribution of an Early Internal Medicine Rotation to the Clinical Reasoning Learning for Young Residents

**DOI:** 10.25122/jml-2020-1003

**Published:** 2020

**Authors:** Silvia Sovaila, Adrian Purcarea, Antoine Froissart, Brigitte Ranque, Pierre Kieffer, Emmanuel Andres, Cecile Goujard, Jean-Christophe Weber, Jean-François Bergmann, Stephane Gayet, Brigitte Granel, Anne Bourgarit

**Affiliations:** 1. Internal Medicine Department, Civil Hospital, University Hospital of Strasbourg, Strasbourg, France; 2. Internal Medicine Department, University Hospital Strasbourg, Strasbourg, France; 3. Internal Medicine Department, Créteil, Assistance Publique – Hôpitaux de Paris, France; 4. Vascular Medicine Department and Reference Center for Rare Vascular Diseases, Hôpital Européen Georges Pompidou, Assistance Publique – Hôpitaux de Paris, France; 5. Internal Medicine Department, Mulhouse hospital, Mulhouse; 6. Internal Medicine Department, Bicêtre Hospital, Assistance Publique – Hôpitaux de Paris, France; 7. Internal Medicine Department, Civil Hospital, University Hospital of Strasbourg, Strasbourg, France; 8. Internal Medicine Department, Hôpital Lariboisiere, Assistance Publique – Hôpitaux de Paris, France; 9. Internal Medicine Department, Hôpital Timone, Marseille, France; 10. Internal Medicine Department, Marseille University Hospitals, Marseille, France

**Keywords:** Clinical reasoning, Script Concordance Tests, internal medicine rotation, residency

## Abstract

Clinical reasoning is the cornerstone of medical practice, and achieving this competence depends on a large number of factors. Internal medicine departments provide junior doctors with plentiful and varied patients, offering a comprehensive basis for learning clinical reasoning.

In order to evaluate the usefulness of an early rotation at internal medicine departments, we compared, via script concordance tests, the evolution of residents’ clinical reasoning after an initial internal medicine rotation compared to rotations through other medical specialties.

Twenty-two residents were tested after six months of their internal medicine rotation and compared to twenty-five residents that had the first rotation in another specialty (control). We showed a significant difference in the improvement of the script concordance tests scores (p=0.015) between the beginning and the end of their first rotation between the internal medicine and the control groups, and this implies the lower improvement of clinical reasoning skills and spontaneous learning slope of the junior doctors in other departments.

## Introduction

Clinical reasoning forms the cornerstone of medical practice. It is acquired in the early years of medical studies and is refined throughout practice, as experience is gained. The intellectual process of medical reasoning is difficult to model because of its complexity [1-3]. So far, the hypothetical-deductive model is generally accepted as the closest to reality [4] and is considered by several authors as a model for clinical reasoning learning and teaching [5].

To assess this specific competence, script concordance testing (SCT) based on both the script theory [6] and the hypothetical-deductive model, have demonstrated their value and their ability to discriminate between different levels of clinical reasoning expertise [7]. SCT measures the capacity to reassess a clinical scenario in conjunction with new information.

Relatively little attention has been paid to how students learn and develop their ability to reason in the face of clinical problems and the factors that influence this kind of learning. Methods such as simulation models, explicit guidance, observation, discussion of clinical cases, and feedback are probably effective methods in clinical reasoning training [8]. On the other hand, the immersion in the responsibilities imposed by the direct care of patients and the need to be confronted with diagnostic problems during the medical residency may be a good time for the crystallization of clinical reasoning ability.

In many countries, before medical specialization, residency curriculum comprises early rotations at internal medicine departments, as the polypathological patient profile allows a complex, varied and comprehensive basis for the learning of clinical reasoning by young doctors.

In order to confirm the usefulness of the first rotation in internal medicine departments rather than other units in medical reasoning training, we evaluated the progression of clinical reasoning, measured by SCT, at the beginning and the end of the rotation in internal medicine departments and compared it to a rotation in other clinical specialties.

## Material and Methods

### Study methods

A multicenter prospective observational study was conducted in ten French internal medicine departments from tertiary (university) and non-university hospitals.

### Study populations

First-year residents that began their first 6-month rotation in one of the ten internal medicine departments in November 2012 and 2013 were proposed to perform the test at the beginning of their first rotation and/or at the end of this first 6-month rotation in an internal medicine department. They were considered as the study population.

The residents were compared with controls that were first-year residents coming in May 2013 and 2014 at the same internal medicine departments after a first 6-month rotation in other specialties.

All participants were included after obtaining signed informed consent. The study was submitted and approved by the ethics committee.

### Tests

The script concordance tests were constructed according to the recommendations of Charlin et al. [9], from 20 most common clinical syndromes encountered in polyvalent internal medicine departments (see supplementary data). A set of 75 questions was sent to 18 experts with over ten years of practice in internal medicine. Sixty questions were selected, with a Cronbach’s alpha value of 0.85. The test rating ranged from 0 to 60, each question weighted score being based on the expert answers with a value between 0 and 1.

### Study

The baseline (Baseline) was considered after the French ranking tests, within the first month after their arrival at the internal medicine departments. As all French students begin residency training at the same time, just after the end of primary formation and French ranking tests, we considered that baseline might not differ between controls and study population.

At the end of an internal medicine rotation, the SCT was conducted in the study population within the last weeks of this 6-month rotation (IM). For those that had already performed the tests at baseline, the order of the test questions was changed. The results of the tests were presented at the end, but not before.

**Figure 1:**
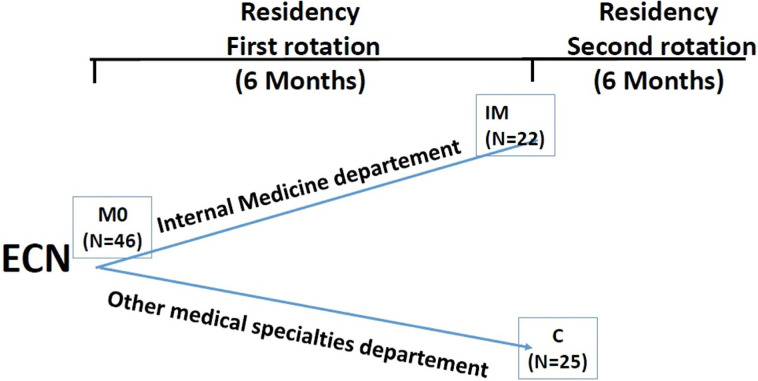
SCT at Baseline (M0), and six months later for the study population (IM) and controls (C).

Controls were tested within the first weeks of their arrival at the internal medicine departments for their second rotation after the first one in other specialties (Control) (Figure 1).

### Statistical analysis

Descriptive statistics are reported as mean and standard deviation.

Since the Baseline, IM and C groups are neither totally independent nor totally dependant, we used the non-parametric Kruskal-Wallis test to compare the variation between the beginning and the end of the training rotations in the study population and the control group.

Cohen’s d was calculated to evaluate the size effect of the internship on the test results.

## Results

The study was conducted in ten internal medicine departments of eight teaching hospitals and two regional hospitals from different regions of France: Ile-de-France (4), Alsace (4), and Provence Alpe Cote d’Azur (2).

### Baseline

**Figure 2:**
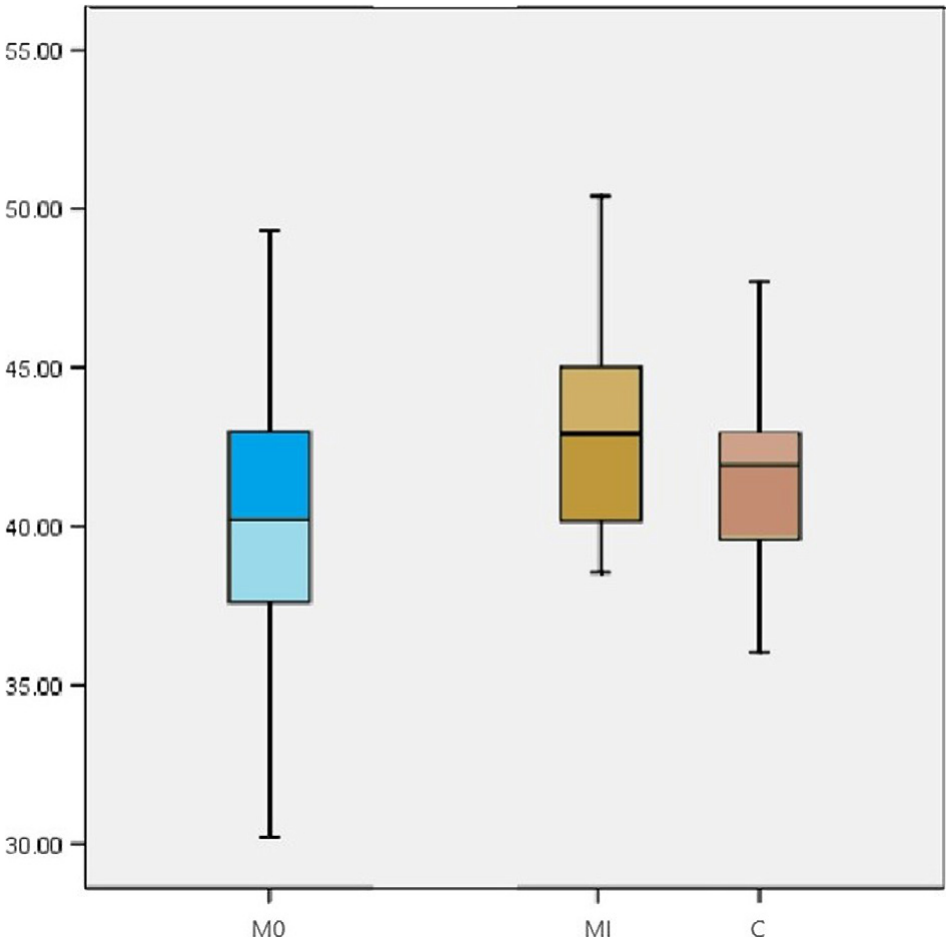
Box SCT results at Baseline (M0), and six months later for the study population (IM) and controls (C).

Forty-six residents, 31 in general medicine and 15 of other medical specialties (rheumatology, dermatology, internal medicine, and others), have completed the test at the beginning of their first 6-month rotation (Baseline), with a mean test score of 40 SD 3.9 (Figure 2). There was no correlation between these results and the National French Board (ENC) rank of these students (rank< 500, n=1; rank 500 to 1000, n=2; rank 1000 to 1500, n= 9, rank 1500 to 8000, n=20).

### 6-months results in the study (IM) and control (C) populations

At the end of their first 6-month rotation in the internal medicine departments (IM), a total of twenty-two residents completed the test: twelve of them had also performed the tests at baseline. The mean test score was 43 SD 3.41 (Figure 2).

The control group that included fifteen residents in general medicine and ten of other medical specialties after a first rotation had a mean SCT score (C) of 42.1 SD 3.5 (Figure 2).

### Comparison of M0, IM and C

As baseline, the IM and C groups were not totally independent, and we used the Kruskal-Wallis non-parametric test to compare groups’ results assuming a progression between groups. This showed a statistically significant difference with a mean rank of 39.3, 58.5, and 51.08 for Baseline, IM, and C, respectively, p <0.02 (Table 1).

**Table 1:** Comparison of SCT group’s results by statistical analysis (Kruskal-Wallis rank test) at Baseline (M0), and six months later for the study population - Internal Medicine (IM) and controls (C), (p<0.015)

	**Different Groups**	**N**	**Mean Rank**
**SCT results**	**Baseline**	46	39
	**Internal Medicine**	22	58
	**Control**	25	51

The calculated value of Cohen’s d of 0.76 corresponded to a strong effect size for the internal medicine traineeship on the test results.

## Discussion

To our knowledge, our study shows for the first time that an early internal medicine rotation has the ability to improve clinical reasoning capacity, measured by SCT and that this improvement is more considerable than after the first rotation in another medical specialty.

The studied population was representative of the normal distribution of residents in internal medicine departments: approximately half comprised medical specialties residents, and the other half included general medicine residents, with a usual repartition from the French National Ranking Exam. This allowed us to consider the baseline results as a baseline for all the resident populations (IM and C).

The SCT is known to be able to assess clinical reasoning under uncertainty [10] and to discriminate between different levels of expertise [11-16]. The SCT validity was ensured by both quantitative (60 tests [17] and a panel of 18 experts [18]) and qualitative criteria (a Cronbach’s alpha value of 0.85) and was confirmed by their discriminatory value between residents and experts [9-11].

Although a six-month rotation is a short time for improving reasoning skills, we were able to show an evolution between the beginning and the end of the internal medicine rotation. This evolution was not that important in the control group. This suggests that a 6-months rotation can improve clinical reasoning skills at the beginning of the residency formation and that internal medicine departments with their comprehensive polypathological and complex patients are probably more performant for this early learning.

The clinical situations tested in our study were all from general internal medicine situations and not rare and/or complex disease cases; therefore, these tests measured the ability to reason more than the internal medicine knowledge. This was confirmed by the lack of correlation between the baseline results for SCT and the French National Ranking Exam.

This study has some limitations. The ideal control group would contain the residents from another specialty at the beginning and the end of their rotation. Since this was not possible, we considered the baseline results of 46 students were representative of baseline of all new residents.

To our knowledge, this is the first study on the variation of the results of SCT in such a short period. Only training levels of one to two years have been studied and showed a significant discriminating power of the SCT [19, 20]. The small differences between our groups might be a reflection of the lack of sensitivity of these tests. Nevertheless, it is also possible that only six months of rotation in internal medicine is not sufficient for clinical reasoning training. It should be noted that in other countries, the internal medicine internship is mandatory on initial training for 1 to 2 years.

Teaching clinical reasoning is difficult, especially in a medical environment with complex interactions, where hierarchy and relationships are not always straightforward [21]. Moreover, the educational role of specialists is outside their area of comfort and not always supported by prior training. It is possible that the clinical reasoning skills improvement was due to the implication of senior medical staff of the participating sites, but also the broad spectrum of clinical pathologies of internal medicine that offers a complete base for learning.

## Conclusion

An early rotation in internal medicine seems to allow a good foundation for clinical reasoning learning at the beginning of the residency. Further studies are needed to confirm our results and to evaluate the exact and specific factors involved in the clinical reasoning learning in young doctors.

## Conflict of Interest

The authors declare that there is no conflict of interest.
